# Sustainable financing to fight HIV/AIDS, TB and malaria: lessons learned from the African Union’s Abuja Declaration

**Published:** 2018-03-01

**Authors:** Mabvuto Kango

**Affiliations:** 1 African Union Commission HQ, Roosevelt road, Addis Ababa, Ethiopia

## Abstract

**Background:**

Investment in malaria control has been proven to contribute to socio-economic development. Concomitant investment in HIV/AIDS and Tuberculosis further augments these socio-economic gains. Africa has used this evidence to guide policy-making, especially for investment in the control of malaria and other infectious diseases. Pursuant to the objective of developing Africa, the Heads of State and Government of the OAU met in 2001 to address the challenges of HIV/AIDS, Tuberculosis and Malaria, a scourge that was ravaging the continent. Noting that the health sector in Africa needed more financial investment, the African leaders adopted a declaration that pledged to allocate at least 15% of their national annual budgets to the health sector.

**Materials and methods:**

Data was collected through review of documents and observations.

**Results:**

The implementation of the Abuja declaration drew a number of mixed results, positive ones which could be scaled up and some challenges that could be used as lessons for improvement.

**Conclusions:**

Taking everything into account, the Abuja Call was a relatively unprecedented success for Africa. With continuous improvement, the initiative could even do better. The African Union should consider revising the Abuja Call, based on lessons learned and emerging issues.

## 1 Introduction

Investment in malaria control has been proven to contribute to socio-economic development [1]. Concomitant investment in HIV/AIDS and tuberculosis further augments these socio-economic gains [2]. Africa has used this evidence to guide policy-making, especially for investment in the control of Malaria and other infectious diseases. The African Union (AU), formerly the Organisation of Africa Unity (OAU), is an intergovernmental organisation of 55 African countries, aimed at fostering integration and socio-economic development. One of Africa’s major hurdles to socio-economic development is the high disease burden, especially HIV/TB and Malaria [3]. The burden does not just arise from the direct consequence of disease, but also from the associated economic costs [1]. Pursuant to the objective of developing Africa, the Heads of State and Government of the OAU met in 2001 to address the challenges of HIV/ AIDS, TB and malaria, a scourge that was ravaging the continent [4].

Noting that the health sector in Africa needed more financial investment, the African leaders adopted a declaration that pledged to allocate at least 15% of their national annual budgets to the health sector. They further committed to make available the necessary resources for the improvement of the comprehensive multi-sectoral response to the three diseases, HIV/AIDS, TB and malaria [5]. The commitment to allocate 15% of national budgets to health was borne out of the realisation that African countries needed to secure adequate financial resources in order to overcome the burden of the three diseases and to harness the economic development that comes with this investment. In addition to the AU declaration, there were other agreements made on health financing at other international fora. For example, the International Taskforce on Innovative Financing recommended an expenditure of at least US$ 34 per person per year on health [6].

However, more than one and half decades later, the aspiration of improving health in Africa with the 15% declaration has not fully turned out as envisioned. Not all countries have managed to meet the target of allocating the 15% of their national budgets to health [7]. So what went wrong? An examination of how the Abuja declaration, sometimes known as the ‘Abuja Call’, was implemented could help to identify challenges, opportunities and lessons learned that could be used to improve future practice. With more technical support for better data generation and development of thinking to match reality, the initiative of the African governments can be effectively supported [8].

## 2 Materials and methods

The objective of the study was to examine the implementation of the Abuja Declaration to allocate 15% of national budgets to health. The methodology employed was a qualitative case study [9] where responses were sought to the questions ‘why’ and ‘how’ was used to get a deeper and better understanding of the performance of the Abuja Declaration. Interviews were undertaken to obtain expert opinions. More secondary data was collected through observations and examination of documents. The two sources of data were used to increase the validity of the collected information [9]. As per well-known approach, most of the data collection was done simultaneously with data analysis [10].

## 3 Results and discussion

The implementation of the Abuja declaration drew a number of mixed results, positive ones which could be scaled up and some challenges that could be used as lessons for improvement. Positive outcomes include the following:

African countries were generally noted to have increased their funds allocated to health following the ‘Abuja declaration’. It was found that at least 27 African countries had increased the proportion of total government expenditures allocated to health [7]. Such kind of increase in health financing was not noted prior to the ‘Abuja declaration’, so it is plausible to attribute this budgetary increase to the influence of the Declaration.

The Abuja declaration led to increased partnerships between governments and other stakeholders. The declaration to allocate 15% of budgets to health was well supported by civil society in Africa. The Africa Public Health Alliance (APHA) led the Civil Society Organisation (CSO) movement on the promotion of the Abuja declaration. In an effort to boost advocacy for the Abuja Declaration, the APHA launched its ‘Africa 15% Campaign’ on 10^th^ December 2006, which was Human Rights Day [11]. The campaign was aimed at encouraging all stakeholders to contribute to the realisation of the Declaration. The campaign’s key message was for governments to allocate 15% of their budgets to health as declared by the Abuja AU Heads of State summit. The APHA went beyond this advocacy and assisted the African Union to regularly collect data on the status of health financing in all African Union Member States. APHA also led the African CSOs in directly lobbying governments. One prominent example of this lobbying was at the 2010 AU Heads of State and Government Summit where they led CSOs into co-signing a letter appealing to African governments to uphold, improve and implement African and global health commitments [12]. APHA also regularly made presentations to high ranking government officials such as Ministers, a practice that was previously considered against protocol in various fora. Collaboration on the ‘Abuja Call’ was extended to the parliamentarians. The APHA facilitated the establishment of the Africa Public Health Parliamentary Network in 2009. The Network was composed of Chairs of African Parliamentary Committees of Health, Gender/ Women, Finance / Budget and Health MDG related issues as outlined above. This stimulated the formation of the African parliamentarians’ platform, were they jointly brainstormed on what they could contribute to the implementation of the ‘Abuja Call’ [13].

The relationship between APHA and African Governments was formed from the commencement of the Abuja Declaration. This was a time when it was believed that African Governments and Civil Society were not getting along in areas of health policy and health financial resources [14]. The relationship between Governments and civil society has been known to be difficult in several sectors [15]. Even in cases were there have been success stories of CSO-Governments collaboration, some challenges have been reported and such relationships take long to blossom [16]. This is one of the unique examples of government cooperation with CSOs especially where it involves sensitive data like financing. It also demonstrates how the Abuja Declaration fostered a fruitful partnership between CSOs and governments in Africa.

Dialogue between Ministries of Health and Ministries of Finance increased. Following the Abuja Declaration, there were a lot of high level follow-up meetings to explore the best ways of implementing the Declaration. Most of these meetings involved high level decision-makers from both Ministries of Health and Ministries of Finance, an occurrence hardly observed prior to the ‘Abuja declaration’. The ultimate outcome was an improvement in dialogue between the two important Ministries, which set the tone for better health financing. The dialogues that took place included the following:

The Annual Meeting of the Harmonization for Health in Africa was hosted in Tunis by the African Development Bank (AFDB) from 1^st^ to 5^th^ December 2009 [17]. At this meeting, senior government officials such as Permanent Secretaries from Ministries in charge of Finance and Health from over a dozen African countries convened to deliberate on the ‘Investment Case for Health Expenditures in Africa’ and to explore strategies to enhance in-country coordination between these two ministries.

Ministers of Health and Ministers of Finance had a dialogue on the margins of the African Union Heads of State and Government Summit in Kampala, Uganda in July 2010 [18]. This meeting included other stakeholders such as the World Bank, The Global Fund and UN Economic Commission for Africa. This meeting identified the reasons for the current poor state of health financing in Africa and defined what could be done by both African and global stakeholders to improve the situation. The meeting also brainstormed on how to strengthen the case for health investment and financing.

A meeting of Ministers of Finance and Ministers of Health was held at the 4^th^ Joint Annual Meetings of the AU Conference of Ministers of Economy and Finance in March 2011 in Addis Ababa. At this meeting, there was a high-level panel discussion on health financing in Africa with the theme ‘More Health for Money and More Money for Health’ [19].

A high-level dialogue between Ministers of Finance and Ministers of Health took place in Tunis, Tunisia, on from 4^th^ to 5^th^ July 2012 under the theme ‘Value for Money, Sustainability and Accountability in the Health Sector’ [20]. The meeting placed emphasis on the urgent need to get the best out of available resources in order to accelerate progress towards meeting the targets of the health Millennium Development Goals (MDGs) and beyond.

During these ministerial dialogues, it was noted from one meeting to another, that the debates between the ministries of finance and health were transforming from defending ones’ ministry to a collective agreement on what should be done to improve health financing. The Abuja Call stimulated these fruitful meetings and should therefore take credit for these.

Creation of The Global Fund for HIV/AIDS, TB and malaria has been partly attributed to the Abuja declaration. Some experts who were in the summit believe that the birth of the Global Fund occurred during the Abuja Summit where the Abuja Declaration was adopted. Within the Abuja Declaration, the African Union Heads of State declared: “*We support the creation of a Global AIDS Fund capitalised by the donor community to the tune of US$5-10 billion accessible to all affected countries to enhance operationalisation of Action Plans, including accessing Anti-retroviral programmes in favour of the populations of Africa*.” [5].

It is this statement by the AU Heads of State and government that is viewed as a key catalyst to the creation of the Global Fund. The Global Fund is a huge and successful initiative that has invested nearly US$4 billion a year since 2002 to affected communities worldwide and its impact has resulted in saving more than 20 million lives worldwide [21]. The support that the Global Fund has provided to Africa is unprecedented.

In the process of implementing the ‘Abuja Call’, some challenges were noted and this has formed the basis for learning for all stakeholders. The lessons learned could go beyond improving the implementation of the Abuja Call itself, as they could also be applicable to other similar initiatives. The learned lessons included the following:

After 10 years of implementation of the Abuja Call, a review [7] found that 27 countries had increased their budgets for health but during the same period, another 11 had reduced their health expenditure. Nine countries were noted to have had no obvious upward or downward movement in their budgets. These findings were out of a total 53 African Union member states.

In the initial dialogues, there were indications that some finance ministries were not keen on other sectors approaching the treasury to dictate or demand what percentage they wanted to receive from the budget. The ministries of finance argued that they already had existing criteria that they used to formulate annual budget and who gets what. One of the possible reasons for this disagreement was that the ministers of finance were not fully represented in (or engaged prior to) the meeting where the Abuja Call was deliberated and adopted.

After ministries of health and the Heads of State agreed at the Abuja summit to allocate 15% of their budget to health, other ministries and sectors also followed the same procedure to adopt declarations allocating themselves certain percentages of the budget. In July 2003, at the African Union summit in Maputo, Mozambique, the heads of state, supported by the ministries of agriculture committed to allocate 10% of national budgets to agriculture [22]. In 2006, at an AU summit, the heads of state, supported by ministries of education made a declaration to allocate 20% of national budgets to education [23]. Once again, ministers of finance were involved but they were not at the centre of creating these later declarations to collate and appropriate or validate all these proportions of the budget.

There is no clear evidence to show that in those countries that attained the 15% target health had improved. The adoption of the 15% budgetary allocation done in Abuja should have been based on a threshold proven to be adequate enough to improve health. A decade after the adoption of the Abuja declaration, it was noted that only 3 countries that had signed up to the declaration were on track to meet MDGs as they were well financed [7].

Most budgets in Africa are relatively small [24], therefore 15% of that budget translates into a meagre amount that could not be significant enough to improve the fight against HIV/AIDS, TB and malaria. It was also noted that governments of 33 countries spent less than US$ 33 per capita on health [5] as opposed to the US$44 recommended by WHO as the minimum [25] and yet some of them had met the 15% Abuja Call target. This shows how meagre the 15% is. Further, total country budget sizes tend to differ from one year to another for various reasons [26] and this entails unpredictable health financing, if it is based on percentage instead of per capita. This could suggest that the decision to adopt a threshold of budgetary allocation calculated in form of percent was not the best option to take.

There were indications from some inter-ministerial conversations that the 15% allocation to health did not clearly indicate whether this was direct allocation to the health sector or it also included investments made in other sectors in charge of socio-determinants of health such as water & sanitation, agriculture, roads, etc. The fund allocators (Ministries of Finance) would sometimes question why ministries of health demanded 15% when the Ministry of Finance had already made investments in other sectors that could lead to improvement of health.

## 4 Conclusions

The Abuja Call did not only bring sensitisation to the importance of increasing funding to the health sector in Africa, but also resulted in increased collaboration between the health sector and ministries of finance and partners such as CSOs. The Abuja Call also stimulated sectors other than health, to lobby for investments into their respective sectors. Taking everything into account, the Abuja Call was a relatively unprecedented success for Africa. With continuous improvement, the initiative could even do better. The African Union should consider revising the Abuja Call, based on lessons learned and emerging issues. The Abuja Call could be revised and be based on per capita expenditure and not percentage of the budget. The percentage allocation is highly dependent on the country’s budget size and therefore does not guarantee the required expenditure on health. Further, total country budget sizes tend to differ from one year to another for various reasons [26] and this entails unpredictable health financing, if it is based on percentage instead of per capita. The per capita allocation should be determined by evidence that dictates that it is an amount adequate enough to improve health.

African countries should only have one benchmark for attaining adequate health financing as opposed to signing up to local benchmarks such as the Abuja Call and then proceeding to commit to other international agreements that have different benchmarks and yardsticks. Further, the amount of money allocated to the health sector should be clearly agreed on whether it is direct support to the health sector or if it includes the investments being made in socio-determinants of health. This could avoid situations where ministries of health would have to justify their funding at every budget allocation exercise in the respective countries. A clear agreement between the ministries of health, finance and ministries in charge of socio-determinants of health could resolve this disagreement.
